# A simulation study for evaluating the performance of clustering measures in multilevel logistic regression

**DOI:** 10.1186/s12874-021-01417-4

**Published:** 2021-11-13

**Authors:** Nicholas Siame Adam, Halima S. Twabi, Samuel O.M. Manda

**Affiliations:** 1 Department of Mathematical Sciences, University of Malawi, Chirunga, Zomba, P.O. Box 280 Malawi; 2 African Institute for Development Policy, Petroda Glasshouse, Area 14, plot number 14/191, Lilongwe 3, 31024 Malawi; 3 Biostatistics Research Unit, South African Medical Research Council, Pretoria, South Africa; 4 Department of Statistics, University of Pretoria, Pretoria, South Africa

**Keywords:** Heterogeneity measures, Clusters, Multilevel logistic regression, Childhood anemia, Binary outcomes

## Abstract

**Background:**

Multilevel logistic regression models are widely used in health sciences research to account for clustering in multilevel data when estimating effects on subject binary outcomes of individual-level and cluster-level covariates. Several measures for quantifying between-cluster heterogeneity have been proposed. This study compared the performance of *between-cluster variance* based heterogeneity measures (the Intra-class Correlation Coefficient (ICC) and the Median Odds Ratio (MOR)), and *cluster-level covariate* based heterogeneity measures (the 80% Interval Odds Ratio (IOR-80) and the Sorting Out Index (SOI)).

**Methods:**

We used several simulation datasets of a two-level logistic regression model *to assess the performance of the four clustering measures for a multilevel logistic regression model.* We also empirically compared the four measures of cluster variation with an analysis of childhood anemia to investigate the importance of unexplained heterogeneity between communities and community geographic type (rural vs urban) effect in Malawi.

**Results:**

Our findings showed that the estimates of SOI and ICC were generally unbiased with at least 10 clusters and a cluster size of at least 20. On the other hand, estimates of MOR and IOR-80 were less accurate with 50 or fewer clusters regardless of the cluster size. The performance of the four clustering measures improved with increased clusters and cluster size at all cluster variances. In the analysis of childhood anemia, the estimate of the between-community variance was 0.455, and the effect of community geographic type (rural vs urban) had an odds ratio (OR)=1.21 (95% CI: 0.97, 1.52). The resulting estimates of ICC, MOR, IOR-80 and SOI were 0.122 (indicative of low homogeneity of childhood anemia in the same community); 1.898 (indicative of large unexplained heterogeneity); 0.345-3.978 and 56.7% (implying that the between community heterogeneity was more significant in explaining the variations in childhood anemia than the estimated effect of community geographic type (rural vs urban)), respectively.

**Conclusion:**

At least 300 clusters with sizes of at least 50 would be adequate to estimate the strength of clustering in multilevel logistic regression with negligible bias. We recommend using the SOI to assess unexplained heterogeneity between clusters when the interest also involves the effect of cluster-level covariates, otherwise, the usual intra-cluster correlation coefficient would suffice in multilevel logistic regression analyses.

## Background

Biomedical research studies often collect binary outcome data that have a multilevel structure. For example, in estimating the efficacy of a new drug on the treatment of patients with a viral infection (recovered or not), patients could be nested within the admitting hospitals. Similarly, for a public health specialist assessing the effect of a feeding intervention on the growth of a child (stunted or not), children could be nested within communities which are also nested within districts. In both examples, patients or children (level-1 units) may have little variation in background characteristics within each hospital or community (level-2 units). On the other hand, hospitals or communities may have patients or children drawn from a wide range of background characteristics, which may affect the within-hospital or within-community homogeneity. A standard logistic regression model, which assumes that the individual outcomes are independent will not consider the hierarchical structure of the data within hospitals or communities, and will consequently fail to distinguish between these two manifestations in each example. Ignoring the dependence between the outcomes may result in an exaggerated number of independent patients or children within hospitals or communities. For the strong homogeneity between patients in hospitals or children in communities, the effect would be an underestimation of the standard errors for the covariate effects, which may lead to incomplete and misleading conclusions on the association between the treatment and the viral infection or the feeding intervention and child growth [1–3].

Multilevel logistic regression models [4], also called hierarchical logistic models [5], are appropriate statistical techniques for analyzing clustered binary outcome data. The use of multilevel models for the analysis of multilevel data structures has exponentiated since the 1990s due to the rise in computational power [2]. The analysis of multilevel data is undertaken mostly to serve two purposes: to provide correct inferences by accounting for the variation that may exist between level-2 units, such as hospitals or communities, and to assess the effects of level-2 risk factors and the unexplained between-cluster (level-2 unit) variation in explaining differences between individuals’ (level-1 units) proneness to the binary outcome [1, 6, 7]. Several techniques in multilevel logistic regression have been proposed to explore whether the individual binary outcomes are explained by cluster-level covariates and the unexplained between-cluster heterogeneity. Because of its simplicity in interpretation, especially in linear mixed models, the Intra-class correlation coefficient (ICC) (also known as the variance partition coefficient (VPC)) is commonly used to measure the unobserved heterogeneity between clusters in multilevel models [8, 9]. In multilevel logistic regression, the ICC loses its natural interpretation which is easily understood and interpreted in the linear mixed model. Thus, several approaches have been developed to provide interpretable and meaningful information regarding the between-cluster heterogeneity and the effect of cluster-level covariates. These methods include the Median Odds Ratio (MOR), the 80% Interval Odds Ratio (IOR-80) and the Sorting Out Index (SOI) [2, 10, 11]. Though they are rarely used, they have nonetheless offered explanations for the unobserved variations between clusters and the contribution of cluster-level predictors on the individual binary outcomes [3, 12].

This paper assessed the performance of the ICC, MOR, IOR-80 and SOI in measuring the importance of cluster-level covariates and unexplained between-cluster heterogeneity in the analysis of a two-level logistic regression model. We compared the performance using simulation studies as well as an empirical application to childhood anemia data from Malawi.

## Two-level logistic regression model

Suppose there are *H* clusters and each cluster has *N*_*h*_ individuals. Let *Y*_*hi*_ be the binary outcome taking a value of 1 or 0 for each individual *i* in cluster *h*. Also, let us assume that the probability of *Y*_*hi*_=1 is *π*_*hi*_, and *π*_*hi*_/1−*π*_*hi*_ is the odds of *Y*_*hi*_=1,(*i*=1,2,…,*N*_*h*_;*h*=1,2,…,*H*). For each individual *hi*, there is a vector *X*_*hi*_ containing values of *p* predictors, including cluster-level covariates. The equation of a two-level logistic regression model is given as

1 $$ log\left(\frac{\pi_{{hi}}}{1-\pi_{{hi}}}\right) =\beta_{0} + \beta_{1}X_{{hi}} + u_{h}   $$

Equation (1) is the conditional logistic model where *π*_*hi*_ is the probability that each individual *i* in cluster *h* takes on the value *Y*_*hi*_=1 and depends on the value of the cluster random effect, *u*_*h*_, which captures the effect of unmeasured cluster-level variability that affects the outcome propensity. They are usually assumed to be normally distributed with a mean of 0 and variance of $\sigma ^{2}_{h}$, and are uncorrelated with the individual and cluster-level predictor variables. In this way, heterogeneity of the binary outcome (*Y*_*hi*_=1) propensity between the clusters would have been accounted for. For example, increasing values of *u*_*h*_ indicate that the associated clusters have higher propensities on the binary outcome (*Y*_*hi*_=1) and greater heterogeneity between the outcomes is implied by higher values of $\sigma ^{2}_{h}$.

### Intra-class correlation coefficient (ICC)

In biomedical research studies, the intra-class correlation coefficient (ICC) statistic is used in multilevel analysis to measure the extent of within-cluster homogeneity, which is caused by the multilevel structure of the data [13, 14]. The statistic is obtained by calculating the ratio of the amount of between-cluster variance to the sum of between- and within-cluster error variances, and it is computed as follows:

2 $$ \rho = \frac{\sigma^{2}_{u}}{\sigma^{2}_{u}+\sigma^{2}_{e}}   $$

where $\sigma ^{2}_{u}$ and $\sigma ^{2}_{e}$ are the variances of the between-cluster and the within-cluster errors, respectively. Since adding the two variances give the total variance of the outcome variable, *ρ* is often interpreted as the proportion of the variance explained by clustering, or as a measure of within-cluster homogeneity. If the value of ICC is 0 then the observed outcomes are completely independent, and if the value is 1 then the outcomes are completely dependent on the cluster they belong to. Thus, the ICC ranges from 0 to 1, and using cut-off points in Koo et al. [13] and Merlo et al. [15], values of ICC of less than 0.50, between 0.50 and 0.75, between 0.76 and 0.90 and greater than 0.90 indicate low, moderate, high and very high correlations, respectively. For the linear mixed model, both the random effect and individual error terms are on the same linear scale, which makes it easier to compute the total variance in the outcome variable. However, for the multilevel logistic regression model, the cluster-level variance is on the logistic scale which cannot be directly understood in terms of variations between clusters in the prevalence of the binary outcome. Furthermore, the individual-level variance is taken from an underlying standard logistic distribution. The value of the variance for this underlying distribution is $\sigma ^{2}_{e}=\pi ^{2}/3=3.29$. Thus, as pointed out in Merlo et al. [12] and Sanagou et al. [3], the usefulness of ICC is somehow limited, largely due to its dependence on linear mixed model concepts regarding partitioning of the total variance, its association with the prevalence of the outcome variable, and the different scales between cluster-level and individual-level error variances. Thus, for public health and epidemiological usages, explaining the partitioning of total variance and ICC for dichotomous outcomes is most challenging.

### The median odds ratio (MOR)

As an alternative to the ICC-based clustering measure in multilevel logistic regression, Larsen and Merlo [10] proposed expressing the between-cluster variance on the odds ratio (OR) scale, which could then be easily interpreted as it is on the same scale on which the effects of predictor variables are measured. For the calculation of MOR, one considers a set of all odds ratios that could be obtained by comparing two individuals with identical covariates but from two different clusters, where one has higher random effect and the other with a lower random effect. The two random effect values are ordered so that the odds ratio is always at least one. The median of these odds ratios provides the Median Odds Ratio (MOR) statistic and, assuming that the random effects are normally distributed, the MOR is given as [10]:

3 $$ {\begin{aligned} \text{MOR}= \exp\left(\sqrt{2\sigma^{2}_{u}} \times \Phi^{-1}(0.75) \right) = \exp\left(\sqrt{2\sigma^{2}_{u}} \times 0.6745\right)\\ = \exp\left(0.95 \times \sqrt{\sigma^{2}_{u}}\right). \end{aligned}}  $$

where *Φ*^−1^(0.75) is the 75th percentile of a standard normal distribution. If $\sigma ^{2}_{u}=0$, then MOR=1, which is indicative of the absence of between-cluster heterogeneity, while if $\sigma ^{2}_{u}$ is large (i.e. considerable between-cluster heterogeneity), the MOR will be much larger than 1 [3, 16]. Thus, the MOR measures heterogeneity between individuals belonging to different clusters.

### Interval odds ratio - 80% (IOR-80)

The interval odds ratio (IOR), as developed in Larsen and Merlo [10] assesses the impact of cluster-level predictor variables on the individual binary outcomes. One starts by considering two different clusters that differ by one unit on a given cluster-level predictor variable. Two individuals with the same values for the remaining cluster-level predictor variables and all the individual-level covariates are chosen, one from each of the two clusters. Then the odds ratio (OR) for the predictor is computed, which often measures the effect of moving to a higher value of the given cluster-level predictor (for a binary cluster-level predictor variable, the OR is just the odds of it being 1 versus 0). Considering all possible pairs of individuals and their ORs, the 80% interval around the median of the distribution of the OR values provides the 80% interval odds ratio (IOR-80). Under the assumption of a normally distributed random effect, the IOR-80 has a lower limit of $\text {IOR}_{L}=\exp (\alpha +\sqrt {(2\times \sigma ^{2}_{u})}\times (- 1.2816))$ and an upper limit of $\text {IOR}_{U}=\exp (\alpha +\sqrt {(2\times \sigma ^{2}_{u})}\times (+ 1.2816))$ [10, 17], and values -1.2816 and +1.2816 represent the 10th and 90th percentiles of the standard normal distribution, respectively. Thus, the limits of the IOR-80 are given as:

4 $$\begin{array}{@{}rcl@{}} \text{IOR}_{L} &=& \exp\left(\alpha - \sqrt{\left(2 \times \sigma^{2}_{u}\right)} \times 1.2816\right), \end{array} $$

5 $$\begin{array}{@{}rcl@{}} \text{IOR}_{U} &=& \exp\left(\alpha + \sqrt{\left(2 \times \sigma^{2}_{u}\right)} \times 1.2816\right), \end{array} $$

where *α* is the regression coefficient for the cluster-level predictor of interest. If the between-cluster variance is small, then the IOR-80% interval will be narrow, and if the between-cluster variance is large, then the IOR-80% interval will be wider. IOR-80% will contain a 1 if the cluster heterogeneity is large compared to the effect of the cluster-level predictor. If the interval will not contain 1, then the effect of the cluster-level predictor (positively or negatively) is larger compared to the unexplained between-cluster variation [10]. Thus, the addition of the cluster-level predictor effect helps to compare the importance of between-cluster heterogeneity and cluster-level predictors in understanding the differences in individual proneness to the outcome, *Y*_*hi*_=1. However, the choice of the 80% interval width is rather arbitrary. Longer or shorter interval widths may or may not contain a 1 when the 80% width actually contains a 1.

### Sorting out index (SOI)

To remedy the problem of IOR depending on the choice of the interval width, Chaix et al. [18] suggested using the percentage in the distribution of the odds ratios as described under the IOR for which the value is greater than 1. Thus, if the cluster residual variance is high, and the effect of the cluster-level predictor is not able to distinguish high-risk clusters from low-risk clusters, then the odds ratios between clusters for the cluster effect would be greater than 1 in half of the cases. On the other hand, if the cluster-level predictor has a huge positive effect compared to the between-cluster heterogeneity, then the odds ratios would be greater than 1 in almost all the pairwise cases of individuals. The percentage range of 50-100% is often used as a *sorting out index* (SOI) to assess and measure the extent to which a given cluster-level covariate is important compared to the between-cluster heterogeneity in explaining the individual proneness to *Y*_*hi*_=1.

The cluster-level covariate could be assumed to be negatively associated with the outcomes, in which case one would formulate the SOI differently. It will still be in half of the cases if it is very weak, otherwise it will be in none of the cases where the odds ratio is greater than 1; then the SOI will range between 0 to 50%. Thus, the percentage range of the SOI can be between 0% and 100% [11, 19]. Assuming the random effect is normally distributed, the sorting out index (SOI) is calculated as:

6 $$ \text{SOI} = \Phi\left(\frac{\alpha}{\sqrt{2\sigma^{2}_{u}}}\right)  $$

The value of *Φ* for $\left (\frac {\alpha }{\sqrt {2\sigma ^{2}_{u}}}\right)$ can be evaluated from a standard normal distribution table and is expressed as a percentage. This information indicates the degree to which the cluster-level predictor is of importance as compared to the unexplained between-cluster heterogeneity. An SOI of near 0% or 100% implies an absence of between-cluster variability and a high negative or positive effect of the cluster-level predictor, respectively. The SOI of 50% implies that the effect of cluster-level predictor is low [19], as compared to the unexplained between-cluster heterogeneity.

## Simulation protocol

We compared the performance of ICC, MOR, IOR-80, and SOI on simulated two-level binary datasets with individual and cluster-level covariates. We then fitted two-level logistic regression models to the datasets and compared the resulting estimates to the true values by the root mean square errors.

For the simulation, the response variable, *Y*_*hi*_, was distributed as a Bernoulli random variable. The following logit model on the probability, *π*_*hi*_=*Pr*(*Y*_*hi*_=1), was used to simulate the binary responses:

7 $$ \text{logit}(\pi_{{hi}})= \beta_{0}+\beta_{1} X_{hi1} +\beta_{2} X_{hi2} + \alpha X_{h} +u_{h}  $$

We assumed the cluster-specific random effect, *u*_*h*_, followed a normal distribution with a mean of 0 and a variance of $\sigma ^{2}_{u}$ (i.e. $u_{h}\sim N(0, \sigma ^{2}_{u})$). The fixed effect parameters were fixed at *β*_0_=0.5,*β*_1_=−1.5,*β*_2_=0.3,*α*=−0.3, and both level-1 covariates *X*_*hp*_∼*N*(0,1) for *p*=1,2, and level-2 covariate *X*_*h*_∼Bernoulli(0.5). For example, to generate 10 clusters with each cluster having 5 individuals with the outcome *Y*_*hi*_=1 or 0, the following steps were implemented;

- We drew 10 random effects, *u*_*h*_, for the 10 clusters distributed from a normal, $u_{h}\sim N(0, \sigma ^{2}_{u})$, for example a value of $\sigma ^{2}_{u}=0.2.$
- Take the first value of the generated random effect, say *u*_*h*_=1.78.
  Repeat the following steps (c-f) 5 times for cluster 1 with the random effect value of 1.78.
- Draw *X*_*hi*1_ from a *N*(0,1) and *X*_*hi*2_ from a *N*(0,1) and *X*_*h*_ from Bernoulli(0.5).
- Substitute the generated values into the equation: logit(*π*_*hi*_)=0.5−1.5*X*_*hi*1_+0.3*X*_*hi*2_−0.3*X*_*h*_+1.78
- We then solve for $\pi _{{hi}}= \frac {1}{1+ \exp {(-(\beta _{0}} + \beta _{1} X_{hi1} + \beta _{2} X_{hi2} + \alpha X_{h}+ u_{h}))}$
- We then draw *Y*_*hi*_ from a Bernoulli with parameter *π*_*hi*_, which results in either 1 or 0 as a binary outcome for subject 1 in the cluster 1.
- Return to step b) and pick the next value, for example -0.568, as the random effect value for cluster 2. This is repeated 10 times to generate 10 clusters with known random effect values, each cluster having 5 individuals.

Here, we fixed the values for $\sigma ^{2}_{u}$ at 0.2, 0.5, 1.0 and 1.5. The resulting true values of ICC, MOR, SOI and IOR-80 are shown in Table 1.

**Table 1:** True values of fixed effects and measures of heterogeneity for the two-level logistic regression, under various random effects variance values

$\sigma ^{2}_{u}$	*β*_0_	*β*_1_	*β*_2_	*α*	ICC	MOR	SOI	IOR-80	IOR-80
								Lower	Upper
0.20	0.5000	−1.5000	0.3000	−0.3000	0.0570	1.532	0.3176	0.3294	1.667
0.50	0.5000	−1.5000	0.3000	−0.3000	0.1319	1.963	0.3821	0.2057	2.669
1.00	0.5000	−1.5000	0.3000	−0.3000	0.2331	2.596	0.4160	0.1209	4.538
1.50	0.5000	−1.5000	0.3000	−0.3000	0.3131	3.216	0.4312	0.0805	6.819

The process was repeated for the varied number of clusters (i.e., H = 10, 50, 100, 300, 500 clusters), number of observations within each cluster (i.e., *N*_*h*_ = 5, 20, 50, 100, 250). For each combination of the number of clusters, cluster size, and between cluster variance, 1000 datasets were simulated. The root mean square error (RMSE) $\sqrt {\frac {(E_{i}-O_{i})^{2}}{N}}$, where *E*_*i*_ was the estimated value and *O*_*i*_ the true value as shown in Table 1, were computed to determine their accuracies. We used the open software R (R Core Team, 2020) and packages *rms* (regression modeling strategies) [20], *lmerTest* and *pbkrtest* in simulating and analyzing the data.

## Simulation results

Tables 2 and 3 present the accuracies of estimates of the fixed effects and measures of heterogeneity for the two-level logistic regression model for a varied number of clusters, cluster sizes and cluster variances. Tables 4 and 5 present results of estimated values of the fixed effects and measures of heterogeneity for the 2-level logistic regression model, under various numbers of clusters, cluster sizes, and cluster variances.

**Table 2:** Average Root Mean Square Errors for the estimated fixed effects, cluster effect, ICC, MOR, IOR-80 and SOI at cluster variances of 0.2 and 0.5

Clusters	Cluster size	*β*_0_	*β*_1_	*β*_2_	*α*	ICC	MOR	SOI	IOR-80	IOR-80
	Size								Lower	Upper
$\mathbf {\sigma ^{2}_{u}=0.2}$										
10	5	0.042	0.002	0.076	0.082	0.034	0.226	0.125	0.063	0.333
	20	0.012	0.008	0.000	0.010	0.003	0.018	0.010	0.007	0.002
	50	0.000	0.003	0.003	0.015	0.002	0.013	0.007	0.011	0.002
	100	0.008	0.002	0.002	0.008	0.002	0.010	0.008	0.001	0.032
	250	0.007	0.027	0.010	0.015	0.004	0.023	0.018	0.003	0.062
50	5	0.041	0.008	0.020	0.045	0.010	0.058	0.037	0.007	0.215
	20	0.020	0.039	0.002	0.011	0.004	0.029	0.018	0.018	0.031
	50	0.011	0.010	0.001	0.040	0.001	0.005	0.053	0.008	0.046
	100	0.028	0.006	0.002	0.050	0.004	0.022	0.022	0.023	0.040
	250	0.065	0.013	0.000	0.028	0.005	0.031	0.029	0.004	0.008
100	5	0.032	0.011	0.034	0.043	0.009	0.054	0.022	0.023	0.010
	20	0.005	0.018	0.012	0.008	0.001	0.008	0.007	0.002	0.016
	50	0.005	0.004	0.003	0.000	0.001	0.008	0.006	0.000	0.002
	100	0.002	0.003	0.009	0.016	0.002	0.010	0.012	0.003	0.038
	250	0.002	0.003	0.002	0.013	0.002	0.015	0.011	0.002	0.053
300	5	0.003	0.005	0.008	0.025	0.011	0.071	0.038	0.022	0.018
	20	0.002	0.009	0.004	0.003	0.001	0.009	0.004	0.004	0.010
	50	0.006	0.001	0.001	0.001	0.001	0.008	0.005	0.002	0.008
	100	0.001	0.005	0.003	0.006	0.001	0.007	0.005	0.001	0.023
	250	0.004	0.002	0.004	0.001	0.001	0.005	0.010	0.002	0.009
500	5	0.011	0.001	0.001	0.000	0.016	0.108	0.097	0.005	0.017
	20	0.006	0.006	0.001	0.001	0.001	0.008	0.058	0.003	0.002
	50	0.006	0.003	0.002	0.006	0.000	0.003	0.055	0.003	0.002
	100	0.002	0.003	0.002	0.016	0.000	0.003	0.050	0.002	0.002
	250	0.019	0.001	0.001	0.009	0.001	0.005	0.053	0.002	0.002
$\mathbf {\sigma ^{2}_{u}=0.5}$										
10	5	0.003	0.000	0.010	0.003	0.003	0.153	0.017	0.036	0.347
	20	0.019	0.012	0.009	0.032	0.003	0.038	0.017	0.002	0.176
	50	0.040	0.010	0.000	0.090	0.003	0.033	0.016	0.000	0.160
	100	0.070	0.010	0.000	0.070	0.008	0.045	0.028	0.024	0.101
	250	0.020	0.000	0.010	0.050	0.003	0.019	0.022	0.014	0.101
50	5	0.000	0.060	0.030	0.060	0.031	0.180	0.018	0.064	0.309
	20	0.010	0.000	0.010	0.030	0.004	0.023	0.015	0.004	0.091
	50	0.020	0.000	0.000	0.030	0.005	0.029	0.019	0.004	0.015
	100	0.020	0.010	0.010	0.000	0.003	0.015	0.009	0.004	0.014
	250	0.020	0.000	0.000	0.030	0.008	0.046	0.013	0.001	0.004
100	5	0.000	0.040	0.010	0.010	0.009	0.052	0.013	0.014	0.059
	20	0.040	0.010	0.020	0.010	0.004	0.025	0.007	0.006	0.051
	50	0.020	0.000	0.000	0.010	0.002	0.010	0.005	0.004	0.011
	100	0.010	0.000	0.000	0.030	0.006	0.032	0.013	0.016	0.001
	250	0.030	0.000	0.000	0.030	0.002	0.014	0.010	0.004	0.051
300	5	0.020	0.000	0.030	0.010	0.008	0.048	0.008	0.006	0.031
	20	0.010	0.000	0.000	0.000	0.004	0.022	0.005	0.004	0.049
	50	0.000	0.010	0.010	0.020	0.002	0.009	0.007	0.006	0.059
	100	0.030	0.000	0.000	0.030	0.002	0.013	0.010	0.004	0.041
	250	0.002	0.001	0.001	0.002	0.001	0.008	0.003	0.000	0.014
500	5	0.010	0.000	0.010	0.010	0.005	0.028	0.008	0.004	0.049
	20	0.000	0.000	0.010	0.000	0.002	0.013	0.002	0.004	0.029
	50	0.000	0.000	0.000	0.020	0.001	0.005	0.007	0.004	0.021
	100	0.004	0.000	0.002	0.014	0.002	0.058	0.006	0.003	0.017
	250	0.008	0.002	0.001	0.031	0.003	0.019	0.012	0.003	0.014

**Table 3:** Average root mean square errors for the estimated fixed effects, cluster effect, ICC, MOR, IOR-80 and SOI at cluster variances of 1.0 and 1.5

Clusters	Cluster	*β*_0_	*β*_1_	*β*_2_	*α*	ICC	MOR	SOI	IOR-80	IOR-80
	Size								Lower	Upper
$\mathbf {\sigma ^{2}_{u}=1.0}$										
10	5	0.062	0.008	0.009	0.010	0.028	0.217	0.011	0.012	0.870
	20	0.120	0.001	0.012	0.202	0.007	0.050	0.056	0.021	0.855
	50	0.042	0.041	0.001	0.024	0.012	0.079	0.017	0.007	0.086
	100	0.094	0.017	0.020	0.167	0.011	0.076	0.040	0.022	0.479
	250	0.002	0.009	0.008	0.096	0.005	0.034	0.031	0.009	0.424
50	5	0.023	0.009	0.002	0.015	0.020	0.135	0.012	0.012	0.478
	20	0.037	0.009	0.003	0.068	0.012	0.081	0.022	0.001	0.531
	50	0.025	0.006	0.001	0.006	0.005	0.036	0.008	0.002	0.117
	100	0.003	0.006	0.000	0.014	0.005	0.036	0.013	0.004	0.001
	250	0.006	0.006	0.005	0.024	0.006	0.039	0.010	0.001	0.228
100	5	0.004	0.004	0.024	0.032	0.012	0.082	0.008	0.012	0.109
	20	0.021	0.006	0.000	0.005	0.005	0.035	0.005	0.003	0.069
	50	0.013	0.006	0.001	0.032	0.003	0.017	0.010	0.003	0.168
	100	0.021	0.005	0.007	0.031	0.005	0.034	0.009	0.005	0.064
	250	0.004	0.010	0.003	0.018	0.003	0.018	0.007	0.001	0.104
300	5	0.014	0.024	0.012	0.038	0.023	0.151	0.017	0.010	0.627
	20	0.009	0.005	0.006	0.003	0.003	0.020	0.004	0.002	0.035
	50	0.005	0.002	0.008	0.010	0.002	0.015	0.009	0.001	0.069
	100	0.021	0.004	0.003	0.026	0.002	0.015	0.009	0.002	0.168
	250	0.009	0.003	0.001	0.013	0.002	0.015	0.009	0.002	0.069
500	5	0.007	0.033	0.004	0.017	0.025	0.165	0.004	0.019	0.058
	20	0.007	0.006	0.004	0.025	0.005	0.033	0.006	0.006	0.004
	50	0.003	0.001	0.002	0.007	0.002	0.010	0.002	0.001	0.003
	100	0.006	0.006	0.006	0.013	0.003	0.023	0.003	0.002	0.001
	250	0.012	0.004	0.001	0.015	0.002	0.011	0.007	0.001	0.001
$\mathbf {\sigma ^{2}_{u}=1.5}$										
10	5	0.063	0.021	0.035	0.076	0.014	0.124	0.017	0.011	0.394
	20	0.005	0.016	0.028	0.140	0.013	0.120	0.030	0.014	0.459
	50	0.002	0.030	0.006	0.171	0.010	0.091	0.039	0.016	0.429
	100	0.002	0.014	0.002	0.014	0.010	0.085	0.010	0.003	0.360
	250	0.065	0.016	0.004	0.134	0.019	0.162	0.033	0.015	0.130
50	5	0.007	0.013	0.007	0.025	0.041	0.334	0.014	0.016	0.381
	20	0.038	0.015	0.001	0.050	0.013	0.112	0.010	0.008	0.045
	50	0.026	0.003	0.007	0.066	0.003	0.069	0.015	0.010	0.177
	100	0.056	0.003	0.007	0.069	0.008	0.069	0.016	0.007	0.144
	250	0.084	0.005	0.002	0.040	0.009	0.080	0.015	0.007	0.012
100	5	0.012	0.045	0.016	0.032	0.027	0.227	0.008	0.015	0.669
	20	0.040	0.009	0.005	0.047	0.007	0.059	0.011	0.006	0.247
	50	0.027	0.011	0.008	0.052	0.006	0.049	0.011	0.006	0.178
	100	0.069	0.001	0.006	0.069	0.004	0.031	0.016	0.006	0.139
	250	0.010	0.002	0.005	0.066	0.003	0.051	0.015	0.006	0.038
300	5	0.021	0.003	0.012	0.034	0.003	0.131	0.007	0.010	0.291
	20	0.021	0.000	0.006	0.026	0.003	0.016	0.007	0.002	0.214
	50	0.018	0.005	0.003	0.012	0.003	0.015	0.003	0.000	0.121
	100	0.013	0.003	0.001	0.014	0.003	0.046	0.004	0.001	0.123
	250	0.009	0.003	0.001	0.013	0.003	0.115	0.009	0.007	0.360
500	5	0.007	0.005	0.001	0.009	0.003	0.178	0.006	0.008	0.040
	20	0.001	0.005	0.000	0.028	0.003	0.039	0.007	0.000	0.035
	50	0.016	0.000	0.003	0.001	0.003	0.024	0.006	0.001	0.040
	100	0.029	0.001	0.001	0.028	0.003	0.013	0.007	0.002	0.019
	250	0.010	0.003	0.072	0.072	0.003	0.060	0.016	0.002	0.011

**Table 4:** Estimated values of fixed effects and measures of heterogeneity for the two-level logistic regression, under random effects variance values 0.2 and 0.5

Clusters	Cluster	*β*_0_	*β*_1_	*β*_2_	*α*	ICC	MOR	SOI	IOR-80	IOR-80
	Size								lower	upper
$\mathbf {\sigma ^{2}_{u}=0.2}$	**True values**:	**0.500**	**-1.500**	**0.300**	**-0.300**	**0.0570**	**1.532**	**0.3176**	**0.3294**	**1.667**
10	5	0.542	-1.502	0.376	-0.382	0.042	1.417	0.209	0.392	1.334
	20	0.488	-1.508	0.300	-0.290	0.056	1.524	0.322	0.337	1.669
	50	0.500	-1.503	0.303	-0.285	0.055	1.519	0.323	0.340	1.665
	100	0.492	-1.498	0.298	-0.292	0.059	1.541	0.324	0.329	1.699
	250	0.507	-1.527	0.290	-0.315	0.054	1.514	0.304	0.333	1.605
50	5	0.539	-1.508	0.320	-0.255	0.067	1.590	0.354	0.322	1.882
	20	0.481	-1.461	0.298	-0.290	0.054	1.508	0.312	0.347	1.636
	50	0.489	-1.510	0.299	-0.261	0.060	1.546	0.344	0.337	1.763
	100	0.528	-1.506	0.302	-0.350	0.061	1.553	0.296	0.306	1.627
	250	0.565	-1.487	0.300	-0.328	0.052	1.501	0.293	0.333	1.559
100	5	0.532	-1.511	0.334	-0.343	0.062	1.559	0.299	0.306	1.657
	20	0.505	-1.518	0.312	-0.293	0.058	1.534	0.322	0.331	1.683
	50	0.505	-1.496	0.297	-0.300	0.057	1.532	0.317	0.329	1.665
	100	0.498	-1.503	0.309	-0.284	0.058	1.537	0.328	0.333	1.705
	250	0.498	-1.497	0.302	-0.313	0.055	1.517	0.306	0.332	1.614
300	5	0.503	-1.505	0.292	-0.325	0.046	1.461	0.281	0.352	1.485
	20	0.498	-1.509	0.296	-0.297	0.056	1.525	0.318	0.333	1.657
	50	0.495	-1.499	0.301	-0.299	0.057	1.528	0.317	0.332	1.659
	100	0.501	-1.495	0.303	-0.306	0.056	1.526	0.313	0.330	1.644
	250	0.496	-1.498	0.296	-0.301	0.057	1.528	0.317	0.331	1.658
500	5	0.489	-1.499	0.299	-0.300	0.041	1.424	0.279	0.384	1.450
	20	0.494	-1.506	0.299	-0.299	0.057	1.531	0.318	0.330	1.666
	50	0.494	-1.503	0.298	-0.294	0.057	1.531	0.321	0.332	1.674
	100	0.499	-1.503	0.298	-0.284	0.057	1.530	0.326	0.335	1.689
	250	0.481	-1.499	0.301	-0.291	0.058	1.533	0.323	0.332	1.683
$\mathbf {\sigma ^{2}_{u}=0.5}$	**True values**:	**0.500**	**-1.500**	**0.300**	**-0.300**	**0.1319**	**1.963**	**0.3821**	**0.2057**	**2.669**
10	5	0.497	-1.500	0.310	-0.297	0.106	1.816	0.367	0.242	2.323
	20	0.519	-1.489	0.291	-3.332	0.125	1.925	0.366	0.207	2.493
	50	0.516	-1.497	0.313	-0.332	0.126	1.930	0.367	0.206	2.509
	100	0.430	-1.490	0.300	-0.230	0.130	1.920	0.410	0.230	2.770
	250	0.480	-1.500	0.290	-0.250	0.130	1.950	0.400	0.220	2.770
50	5	0.500	-1.440	0.330	-0.240	0.100	1.780	0.390	0.270	2.360
	20	0.490	-1.500	0.290	-0.270	0.130	1.960	0.390	0.210	2.760
	50	0.480	-1.500	0.300	-0.270	0.140	1.990	0.400	0.210	2.820
	100	0.480	-1.510	0.310	-0.300	0.130	1.950	0.380	0.210	2.650
	250	0.520	-1.500	0.300	-0.270	0.120	1.920	0.390	0.220	2.630
100	5	0.500	-1.460	0.290	-0.290	0.130	1.930	0.380	0.220	2.610
	20	0.540	-1.510	0.280	-0.310	0.140	1.990	0.380	0.200	2.720
	50	0.480	-1.500	0.300	-0.290	0.130	1.960	0.390	0.210	2.680
	100	0.510	-1.500	0.300	-0.330	0.140	1.990	0.370	0.190	2.670
	250	0.470	-1.500	0.300	-0.270	0.130	1.950	0.390	0.210	2.720
300	5	0.520	-1.500	0.330	-0.310	0.140	1.990	0.380	0.200	2.700
	20	0.490	-1.500	0.300	-0.300	0.130	1.940	0.380	0.210	2.620
	50	0.500	-1.490	0.290	-0.320	0.130	1.960	0.380	0.200	2.610
	100	0.470	-1.500	0.300	-0.270	0.130	1.950	0.390	0.210	2.710
	250	0.498	-1.499	0.299	-0.302	0.131	1.960	0.381	0.206	2.656
500	5	0.510	-1.500	0.290	-0.290	0.130	1.940	0.380	0.210	2.620
	20	0.500	-1.500	0.310	-0.300	0.130	1.950	0.380	0.210	2.640
	50	0.500	-1.500	0.300	-0.280	0.130	1.970	0.390	0.210	2.720
	100	0.504	-1.501	0.298	-0.286	0.132	1.963	0.388	0.209	2.706
	250	0.508	-1.499	0.299	-0.269	0.131	1.959	0.394	0.213	2.743

**Table 5:** Estimated values of fixed effects and measures of heterogeneity for the two-level logistic regression, under random effects variance values 1.0 and 1.5

Clusters	Cluster	*β*_0_	*β*_1_	*β*_2_	*α*	ICC	MOR	SOI	IOR-80	IOR-80
	Size								lower	upper
$\mathbf {\sigma ^{2}_{u}=1.0}$	**True values**:	**0.500**	**-1.500**	**0.300**	**-0.300**	**0.2331**	**2.596**	**0.4160**	**0.1209**	**4.538**
10	5	0.562	-1.508	0.291	-0.290	0.259	2.800	0.424	0.109	5.408
	20	0.620	-1.501	0.312	-0.502	0.231	2.581	0.361	0.100	3.683
	50	0.542	-1.459	0.300	-0.276	0.230	2.575	0.421	0.128	4.624
	100	0.594	-1.517	0.320	-0.467	0.243	2.669	0.376	0.099	4.059
	250	0.502	-1.509	0.308	-0.396	0.231	2.580	0.389	0.112	4.114
50	5	0.477	-1.509	0.299	-0.315	0.213	2.461	0.407	0.133	4.060
	20	0.537	-1.491	0.297	-0.368	0.221	2.515	0.394	0.120	4.007
	50	0.475	-1.506	0.301	-0.306	0.229	2.565	0.413	0.123	4.421
	100	0.503	-1.494	0.300	-0.287	0.230	2.572	0.419	0.125	4.539
	250	0.495	-1.494	0.295	-0.324	0.228	2.558	0.408	0.122	4.310
100	5	0.522	-1.504	0.324	-0.268	0.222	2.519	0.423	0.133	4.430
	20	0.521	-1.494	0.300	-0.295	0.229	2.568	0.417	0.124	4.469
	50	0.513	-1.494	0.299	-0.332	0.232	2.588	0.407	0.118	4.370
	100	0.501	-1.505	0.307	-0.331	0.236	2.616	0.408	0.116	4.474
	250	0.496	-1.491	0.297	-0.318	0.232	2.586	0.411	0.120	4.434
300	5	0.514	-1.476	0.312	-0.338	0.211	2.445	0.399	0.131	3.911
	20	0.509	-1.505	0.294	-0.297	0.231	2.581	0.416	0.123	4.503
	50	0.496	-1.498	0.292	-0.290	0.234	2.602	0.419	0.122	4.607
	100	0.479	-1.504	0.303	-0.274	0.235	2.608	0.424	0.123	4.706
	250	0.491	-1.503	0.301	-0.287	0.234	2.618	0.417	0.123	4.607
500	5	0.494	-1.467	0.304	-0.283	0.209	2.433	0.415	0.140	4.080
	20	0.493	-1.506	0.304	-0.275	0.228	2.563	0.422	0.127	4.542
	50	0.497	-1.501	0.302	-0.293	0.233	2.595	0.418	0.122	4.565
	100	0.506	-1.494	0.306	-0.287	0.230	2.574	0.419	0.125	4.524
	250	0.489	-1.504	0.301	-0.285	0.235	2.607	0.420	0.122	4.643
$\mathbf {\sigma ^{2}_{u}=1.5}$	**True values**:	**0.500**	**-1.500**	**0.300**	**-0.300**	**0.3131**	**3.216**	**0.4312**	**0.0805**	**6.819**
10	5	0.437	-1.521	0.335	-0.224	0.307	3.166	0.448	0.092	7.213
	20	0.505	-1.484	0.272	-0.440	0.326	3.331	0.403	0.066	6.360
	50	0.498	-1.530	0.294	-0.129	0.314	3.229	0.471	0.097	6.248
	100	0.502	-1.486	0.302	-0.314	0.304	3.143	0.427	0.083	6.459
	250	0.435	-1.516	0.304	-0.166	0.315	3.245	0.464	0.096	6.809
50	5	0.507	-1.487	0.307	-0.325	0.273	2.885	0.418	0.097	5.438
	20	0.538	-1.515	0.300	-0.350	0.324	3.314	0.422	0.073	6.864
	50	0.526	-1.503	0.307	-0.235	0.305	3.147	0.445	0.090	6.996
	100	0.556	-1.503	0.307	-0.369	0.321	3.285	0.418	0.073	6.675
	250	0.416	-1.495	0.298	-0.260	0.304	3.139	0.439	0.088	6.807
100	5	0.488	-1.455	0.284	-0.268	0.286	2.989	0.434	0.096	6.150
	20	0.460	-1.491	0.305	-0.253	0.311	3.198	0.442	0.086	7.066
	50	0.527	-1.511	0.308	-0.352	0.318	3.258	0.420	0.075	6.641
	100	0.432	-1.499	0.306	-0.231	0.311	3.200	0.447	0.087	7.258
	250	0.510	-1.498	0.296	-0.235	0.311	3.197	0.446	0.087	7.201
300	5	0.479	-1.503	0.312	-0.266	0.298	3.085	0.437	0.091	6.528
	20	0.479	-1.501	0.306	-0.275	0.314	3.223	0.437	0.082	7.033
	50	0.518	-1.495	0.297	-0.312	0.312	3.206	0.428	0.080	6.698
	100	0.487	-1.503	0.299	-0.314	0.312	3.208	0.428	0.080	6.696
	250	0.491	-1.503	0.301	-0.287	0.300	3.101	0.432	0.088	6.459
500	5	0.507	-1.495	0.301	-0.309	0.292	3.042	0.426	0.089	6.079
	20	0.499	-1.505	0.300	-0.328	0.309	3.178	0.424	0.080	6.484
	50	0.516	-1.500	0.298	-0.299	0.312	3.202	0.431	0.081	6.779
	100	0.529	-1.501	0.301	-0.328	0.313	3.216	0.425	0.078	6.631
	250	0.510	-1.497	0.228	-0.228	0.310	3.192	0.447	0.088	7.225

### Accuracy of estimated parameters

We observe that the estimated parameters for the fixed effects were accurate for all cluster-levels and cluster variances, particularly for large clusters and cluster sizes. Lower root mean square errors were particularly observed for larger clusters, providing evidence of unbiasedness for the estimates $\hat {\beta }$’s and $\hat {\alpha }$ for increased clusters and cluster sizes as shown in Tables 2 and 3. The RMSEs for $\hat {\beta }_{0}$ were slightly biased for 10 clusters with a cluster size of 5. However, the estimates were unbiased for at least 10 clusters with a cluster size of at least 20 for all cluster-level variances. At a cluster variance of 1.0, the estimates for $\hat {\beta }_{0}$ were unbiased for at least 100 clusters with a cluster size of at least 5. Lower RMSEs for $\hat {\beta _{1}}$ were observed for at least 10 clusters with a cluster size of at least 5 for all cluster variances. Bias for $\hat {\beta _{1}}$ was negligible for 100 clusters with cluster size of at least 50 at a cluster variance of 0.5. At a cluster variance of 0.2, $\hat {\alpha }$ was slightly biased for 10 clusters with a cluster size of 5. However, we observe unbiased estimates of *α* for 100 clusters with cluster sizes of 50, and 500 clusters with a cluster size of at least 5. The bias of $\hat {\alpha }$ improved with the increase in clusters and cluster size for all cluster variances.

### Performance of estimated heterogeneity measures

#### Intra-class correlation coefficient (ICC)

Tables 2 and 3 show that the estimates for the ICC were less biased when the cluster variance was 0.2 and the estimates of the ICC were unbiased with 100 clusters and clusters sizes of 20 or more. At the cluster variance of 0.5, the ICC was estimated with less bias for at least 100 clusters of sizes 50 or larger. At level-2 variance of 1.0, the ICC was slightly biased for 10 clusters with cluster size of 5. However, the bias for estimating the ICC decreased for at least 50 clusters with cluster size of at least 20. At a level-2 variance of 1.5, the lowest RMSEs were observed for the estimation of ICC with at least 10 clusters each of cluster size of at least 20. Increasing the number of clusters (at least 100) and cluster size (at least 20) gave negligible bias for the estimation of ICC as its RMSEs decreased to almost zero, for all level-2 variances.

#### Median odds ratio (MOR)

As observed from Tables 2 and 3, the estimates of the MOR were largely biased for 50 clusters with cluster size of 5, for all cluster variances. at a cluster variance of 0.2, the estimates of the MOR were less biased for at least 50 clusters with cluster sizes of at least 20. The estimates of the MOR improved when cluster sizes were 50 or more for all cluster-level variances. The lowest RMSEs were observed for at least 300 clusters and a cluster size of at least 50 in estimating the MOR.

#### 80% interval odds ratio (IOR-80)

The estimates for the IOR-80 were less accurate for small clusters and cluster sizes when the cluster variance was large as observed from Tables 2 and 3. At a cluster variance of 0.2, we observe low RMSEs when estimating the IOR-80 lower and upper limits for at least 10 clusters with a cluster size of at least 20. At a cluster variance of 0.5, the estimates of the IOR-80 lower limit were accurate for at least 10 clusters with a cluster size of at least 20. However, the estimates for the IOR-80 upper limit were largely biased for at least 10 clusters with a cluster size of at least 5 for cluster variances of 1.0 and 1.5. On the contrary, the estimates for the IOR-80 lower limit at these cluster variances (1.0 and 1.5) were accurate for at least 10 clusters with a cluster size of at least 20. The accuracy of the IOR-80 estimates were less biased with increased clusters and cluster sizes. At a cluster variance 0.2, unbiased estimates of the IOR-80 lower and upper limits were observed for 100 clusters with cluster size of at least 50. Overall, the bias for the estimates of the I0R-80 decreased for at least 300 clusters with a cluster size of at least 100 at all cluster variances.

#### Sorting out index (SOI)

As seen from Tables 2 and 5, the SOI estimates were biased for 10, 50, and 100 clusters with a cluster size of 5. However, the estimates of SOI were less biased for at least 10 clusters with cluster sizes of at least 20 at cluster variances of 0.2 and 0.5. Furthermore, the estimates for SOI were slightly biased for 10 clusters with a cluster size of 5. The bias in estimating the SOI decreased with increased clusters (at least 300) and a cluster size (at least 50) at cluster-level variances of 0.2 and 0.5. In addition, for large cluster variances of 1.0 and 1.5, the estimates of the SOI were unbiased with increased clusters (at least 100) and cluster size (at least 50).

## Application to childhood anemia in Malawi

For the application, we analyzed childhood anemia among under-five children in Malawi using the 2015-16 Malawi Demographic and Health Survey (MDHS) data by fitting a two-level logistic regression model taking the community as a level-two unit. Childhood anemia is a major public health concern in Sub-Saharan Africa (SSA), particularly in Malawi [21]. We categorized anemia among children aged 6 to 59 months who were born five years before the survey as: no anemia = 0 and had anemia = 1.

There were 4676 children aged 6 to 59 months distributed across 850 enumeration areas (EA) which we regarded as communities. The overall prevalence of anemia was 63.1% (95% CI: 61.7, 64.5), with an average prevalence of childhood anemia of 62.2%, with a range of 0.0% to 100% and mode of 100%. The median anemia prevalence across the communities was 66.7%. The average number of children per community was 5.5, with a range of 1 to 15 and mode of 6. The median number of children per community was 5.

There are several factors that have been found to be associated with childhood anemia. These include individual-level factors: age, gender of a child, mother’s age, birth rank, wealth index, mother’s education level, father’s education level, household size, household hunger status, sanitation services, water and electricity supply, and community-level factors: community geographic type and community development index [21–25]. For our application, we used mother’s education, mother’s age, child gender, household wealth quantile, and community geographic type (urban/rural). For the unadjusted model, the estimate of the between-community variance was 0.497, translating to an ICC of 0.131 and an MOR of 1.954. Adjusting for the child-level and the community-level covariates had the following odds ratios on child anemia: being female (OR= 0.92, 95% CI: 0.80, 1.04), being in a household with medium (OR= 0.71, 95% CI: 0.59, 0.84) and rich (OR= 0.78, 95% CI: 0.66, 0.93) wealth, having mothers with primary (OR= 0.69, 95% CI: 0.56, 0.86) and post-secondary (OR= 0.64, 95% CI: 0.49, 0.82) education, and residing in a rural community (OR= 1.21, 95% CI: 0.97, 1.52). For the covariate-adjusted model, the estimate of the between-community variance was 0.455. This translates to estimated values of ICC and MOR to be 0.122 and 1.898, respectively. The estimate of the ICC show that the unexplained heterogeneity between communities (i.e. low homogeneity in childhood anemia in a community) was low. While that of the MOR shows a high level of unexplained community heterogeneity (MOR »1). The estimates of the SOI was 0.567 (translated as 56.7%) and of the IOR-80 was (0.345, 3.978), implying that the unexplained heterogeneity between communities was more significant in explaining a child’s propensity to be anemic than the effect of the community geographic type (rural vs urban).

## Discussion

Multilevel logistic regression models, which recognize the fact that level-1 units (individuals) are nested within higher-level units (clusters) when estimating the effect of covariates at different levels on individual binary outcomes, are widely implemented in most standard statistical software packages. They measure and evaluate the relative variation in the outcome measures, between individuals within and between clusters. However, measuring residual cluster-level variation and covariate effect heterogeneity or homogeneity (clustering) of binary outcomes within high-level clusters is more challenging in multilevel logistic regression than for linear mixed models. We set out to compare the performance of four measures of clustering in a two-level nested logistic regression model through a simulation study. The four measures were ICC, MOR, SOI and IOR-80. We applied the four measures to an analysis of childhood anemia in Malawi using a two-level logistic regression model, where the level-2 units were communities.

The accuracy of the estimated coefficients for the two-level logistic regression model improved for 100 to 500 clusters with cluster sizes of 50 to 250 for all cluster variances. These findings are similar to results from previous studies [26, 27], which showed that as the number of clusters and cluster sizes increased, the accuracy of the regression coefficients had a negligible bias. The estimates of ICC were more accurate than MOR, SOI and IOR-80 when the cluster number was small (at least 10) and when the cluster size was small (at least 20), and the bias for the ICC was negligible when the number of clusters and the cluster sizes increased. This is similar to a study by Moineddin et al. [26] which found that increasing the number of clusters and cluster sizes improved the convergence rate for the ICC.

The estimates of the SOI were more accurate than the estimates for the MOR and the IOR-80 for smaller number of clusters (at least 10) and a cluster size of at least 20 regardless of the cluster variance. However, the estimates of all the measures were more accurate when the number of clusters increased (from 100 to 500) regardless of cluster size and cluster variance. The estimates for MOR and IOR-80 performed better for large clusters (at least 300) and large cluster size (at least 20) at larger cluster variance (1.0 and 1.5). Overall the ICC and SOI estimates were better than the estimates of the MOR and the IOR-80 and the estimation of the heterogeneity between clusters using ICC and SOI was more accurate. However, the ICCs usefulness is limited as it does not account for covariates at the cluster level. Hence, the SOI would be the best statistic to measure the strength of clustering as compared to the ICC, MOR and IOR-80, especially when between cluster-level covariate heterogeneity is also of interest.

In the application to childhood anemia in Malawi, accounting for the purported risk factors, the estimated values of the ICC, and MOR were 0.122 and 1.898, respectively, which were indicative of low similarity between the status of anemia in children within a community, and high variability in unexplained heterogeneity between the communities. The estimates of SOI and IOR-80 were 56.7% and (0.345, 3.978), respectively, which showed that unexplained heterogeneity between communities was of great significance in the understanding of anemia in the children than the child and community-level predictors, namely mother’s education, mother’s age, child sex, community geographic type and household wealth quantile, used in the model, and community-level geographic type (rural/urban). [10]. Mother’s age, wealth quintile and mother’s education were significantly associated with childhood anemia, similar to findings from previous studies [21, 23, 24]. However, the effect of community geographic type on childhood anemia was not significant. With 850 communities, an average of 5 children per community and the simulation study, we recommend using the ICC and SOI to measure the strength of heterogeneity between the communities. However, since the ICC does not take into account the community-level covariate (community geographic type), we recommend using the SOI to measure the strength of variation between communities for this application.

## Conclusion

In conclusion, measures of the between-cluster heterogeneity and effects of cluster-level covariates in multilevel logistic regression models would be estimated with negligible bias when the data has at least 300 clusters with a cluster size of at least 50. The estimation of the intra-class correlation coefficient and the sorting out index are accurate with 10 clusters and a cluster size of 20. We recommend using the sorting out index to assess unexplained heterogeneity between clusters as well as the effect of cluster-level covariates. The usual intra-cluster correlation coefficient would suffice when only quantification of the between-cluster variation is the purpose of the multilevel logistic regression analysis.

## Data Availability

The dataset used in this study are available from the DHS website https://dhsprogram.com/Data/ upon request from the MEASURE DHS program team.
